# Shotgun Metagenomics Reveals *Bacteroides stercoris* as a Fecal Biomarker Depleted in Late-Stage Hepatocellular Carcinoma

**DOI:** 10.1016/j.gastha.2024.07.020

**Published:** 2024-08-30

**Authors:** Damien Esparteiro, Grégory Fouquet, Anoïsia Courtois, Mickaël Naassila, Eric Nguyen-Khac, Ingrid Marcq

**Affiliations:** 1 GRAP INSERM U1247, Amiens, France; 2 Service d’Hépato-Gastro-Entérologie, CHU d’Amiens, Amiens, France

Simultaneous study of the gut and the liver is promising for a better understanding of hepatic malignancies due to their bidirectional communication through the gut-liver axis. Gut Microbiota (GM), which under normal circumstances contributes to the host homeostasis, may become dysbiotic, which can in turn contribute to liver inflammation.^1^ Prolonged liver inflammation in cirrhotic patients, for example due to gut-liver translocation and/or alcohol exposure, can lead to the development of Hepatocellular Carcinoma (HCC), the most frequent form of primary liver cancer, which is associated with a poor prognosis especially since it is often diagnosed at a stage too advanced to be curable. In this study, we characterized the GM of 51 HCC patients included in the prospective French CHIEF (Cohorte prospective de patients atteints de Carcinome Hépatocellulaire en France) cohort using shotgun metagenomics in order to elucidate how the GM changes alongside HCC progression.

Patients were retrospectively selected from the cohort of the Amiens University Hospital based on the availability of their stool samples and the Barcelona Clinic Liver Cancer (BCLC) of the liver biopsy at the inclusion. We regrouped the patients into two groups depending on their BCLC stage; the early-stage group and the late-stage group. 18 and 16 HCC patients with respectively BCLC A and BCLC B stage were included in the early-stage group, and 15 and 2 HCC patients with respectively BCLC C and BCLC D were included in the late-stage group. DNA was extracted from the stool samples (QIAmp Fast DNA Stool Mini Kit, Qiagen) and was then sequenced (NextSeq 550, Illumina). After sequence cleaning, taxonomic assignment was performed using MetaPhlAn 4.^2^ Read counts were analyzed at the species level.

Species richness evaluated using Chao1 index was significantly reduced in late-stage HCC patients (Figure A, 120.9 species for early-stage vs 80.2 species for late-stage, *P* = .005, Welch's t-test). We then tested whether overall GM composition differed between early- and late-stage HCC patients by analyzing beta-diversity using Permutational Multivariate Analysis of Variance (PERMANOVA) of the Bray-Curtis index (Figure B). BCLC staging did not significantly explain GM variability (R^2^ = 0.02, *P* = .289). Thus, in spite of a GM encompassing fewer species for late-grade HCC patients, the GM was overall similar for early- and late-grade HCC, suggesting that the difference in species richness is due to the disappearance of several rare rather than prevalent and abundant species. Then, each species with a prevalence higher than 10% was tested for differential abundance analysis using multivariate linear mixed model with MaAsLin 2^3^ adjusting for age, sex, HCC stage, and etiology. While no species was significantly associated with age, sex or etiology, only one species had a significantly different abundance between early- and late-stage HCC patients, *Bacteroides stercoris,* which was depleted in the GM of HCC patients with an advanced stage (Benjamini-Hochberg corrected *P* = .007). While it is not uncommon in GM analysis to detect significant associations with clinical features, this result is intriguing because of the importance of the difference between early- and late-stage HCC patients, and the fact that no other species was differently abundant. *B. stercoris* represented a non-negligible fraction of the GM of early-grade patients, accounting on average for 0.7% of their GM (Figure C). More strikingly, while *B. stercoris* was detected in stool samples of 22 (64.7%) early-stage HCC patients, it was detected in stool samples of 1 (5.9%) late-stage HCC patient, with a relative abundance below 0.01% (Figure D). Put together, these results suggest that *B. stercoris* can be used as a novel non-invasive fecal biomarker to evaluate HCC progression.

**Figure:**
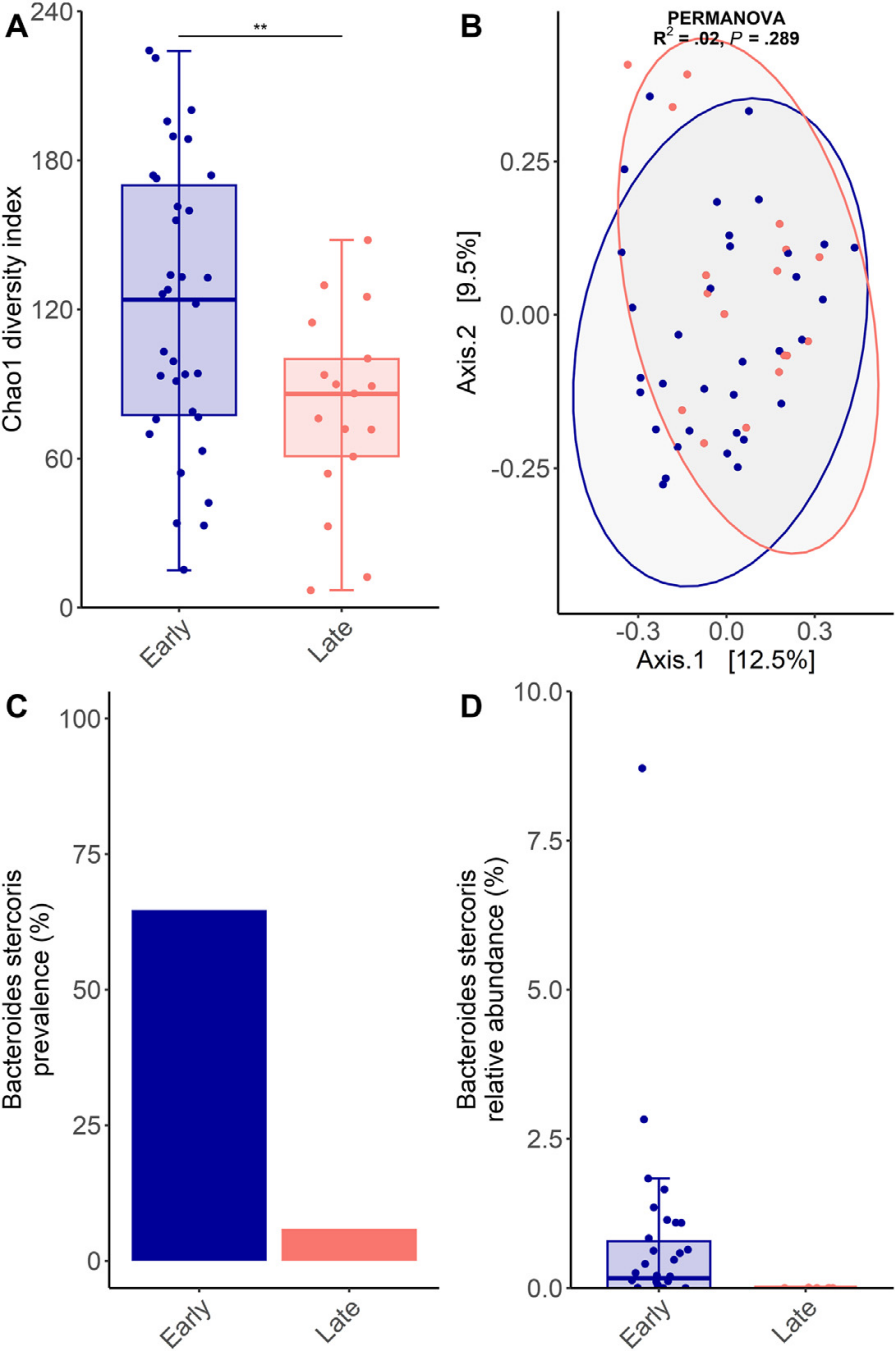
Analysis of the GM of HCC patients. 34 patients were classified as early-grade and 17 patients as late-grade. Alpha-diversity was evaluated using Chao1 index, which was significantly reduced in late-grade HCC (120.9 vs 80.2 species, *P* = .005*,* Welch's t-test (A). However, no difference was found according to a permutational multivariate analysis of variance of the Bray-Curtis beta-diversity metric (B), which suggests that the GM is nonetheless close between early and late-grade HCC. This species was detected in most early-grade HCC patients but only in one late-grade HCC patient (C). Multivariate differential abundance analysis revealed that *Bacteroides stercoris* was significantly enriched in stool samples of early-grade HCC patients (Benjamini-Hochberg corrected *P =* .007).

While past research already characterizing the GM of HCC patients using 16S sequencing did not report *B. stercoris* disappearance in late-stage HCC, the use of metagenomics allowed a taxonomical resolution at the species level that could not be achieved by others.^4^^,^^5^ Another metagenomics study reported that this species was depleted in stool samples of colorectal cancer patients,^6^ while Zaidi *et al.* found that *B. stercoris* was depleted in the circulating microbiota of patients with esophageal adenocarcinoma.^7^ In contrast, a recent study reported that *B. stercoris* was significantly enriched in stool samples of patients with esophageal squamous cell carcinoma, a second subtype of esophageal cancer, in comparison with healthy controls.^8^ Interestingly, an enrichment in the fecal abundance of the same species was detected in patients with advanced predominantly cutaneous melanoma that responded to programmed cell death protein 1 blockade.^9^ Thus, since *B. stercoris* isolation from feces was reported in 1986, this species has been associated with digestive cancers in several studies. On the other hand, only one study provides insight into *B. stercoris* metabolism both in an *in vitro* and in a preclinical model. Ryu *et al.* indeed reported that the culture supernatant of a strain of *B. stercoris* demonstrated an anti-obesity activity *in vitro* and that the oral administration of this strain to mice on high-fat diet significantly reduced weight gain compared to controls, in spite of identical food intake.^10^ The link between this study and the recurrent association of *B. stercoris* with cancers remains unclear.

In summary, we provide in this study evidence that *B. stercoris* represents a potential novel non-invasive biomarker of HCC progression and that this biomarker deserves further evaluation in other studies using multicentric and independent cohorts. In addition, further research is needed to provide a mechanistic explanation about the depletion of *B. stercoris* in the GM of late-stage HCC patients, and more generally its possible role in digestive cancers.
